# 免疫检查点抑制剂相关消化系统不良反应的临床诊治建议

**DOI:** 10.3779/j.issn.1009-3419.2019.10.10

**Published:** 2019-10-20

**Authors:** 玥 李, 汉萍 王, 潇潇 郭, 佳鑫 周, 炼 段, 晓燕 斯, 丽 张, 小伟 刘, 家鸣 钱, 力 张

**Affiliations:** 1 100730 北京，中国医学科学院，北京协和医学院，北京协和医院消化内科 Department of Gastroenterology, Peking Union Medical College Hospital, Chinese Academy of Medicine Sciences & Peking Union Medical College, Beijing 100730, China; 2 100730 北京，中国医学科学院，北京协和医学院，北京协和医院呼吸内科 Department of Respiratory Medicine, Peking Union Medical College Hospital, Chinese Academy of Medicine Sciences & Peking Union Medical College, Beijing 100730, China; 3 100730 北京，中国医学科学院，北京协和医学院，北京协和医院心内科 Department of Cardiology, Peking Union Medical College Hospital, Chinese Academy of Medicine Sciences & Peking Union Medical College, Beijing 100730, China; 4 100730 北京，中国医学科学院，北京协和医学院，北京协和医院风湿免疫科 Department of Rheumatology and Immunology, Peking Union Medical College Hospital, Chinese Academy of Medicine Sciences & Peking Union Medical College, Beijing 100730, China; 5 100730 北京，中国医学科学院，北京协和医学院，北京协和医院内分泌科 Department of Endocrinology, Peking Union Medical College Hospital, Chinese Academy of Medicine Sciences & Peking Union Medical College, Beijing 100730, China; 6 100730 北京，中国医学科学院，北京协和医学院，北京协和医院检验科 Department of Clinial of Laboratory, Peking Union Medical College Hospital, Chinese Academy of Medicine Sciences & Peking Union Medical College, Beijing 100730, China; 7 100730 北京，中国医学科学院，北京协和医学院，北京协和医院眼科 Department of Ophthalmology, Peking Union Medical College Hospital, Chinese Academy of Medicine Sciences & Peking Union Medical College, Beijing 100730, China

**Keywords:** 免疫检查点抑制剂, 免疫相关不良反应, 肝脏毒性, 消化道不良反应, Immune-checkpoint inhibitors, Immune-related adverse events, Liver toxicity, Gastrointestinal adverse events

## Abstract

恶性肿瘤的免疫治疗是当前肿瘤研究和治疗领域的热点，免疫检查点分子程序性死亡受体-1（programmed cell death receptor-1, PD-1）和细胞毒性T淋巴细胞相关抗原4（cytotoxic T lymphocyte-associated antigen 4, CTLA-4）相关信号通路的激活可以抑制T淋巴细胞活化，肿瘤细胞通过激活该信号通路实现免疫逃逸。免疫检查点抑制剂（immuno-checkpoint inhibitors, ICIs）通过抑制该信号通路，活化T淋巴细胞发挥机体对肿瘤细胞的清除。因此，ICIs的相关毒性包括免疫相关的不良事件（immune-related adverse effects, irAEs）。消化系统如胃肠道、肝脏作为人体重要的消化吸收器官、代谢解毒器官，同时也是重要的免疫相关器官，是irAEs的常见受累系统。本文将分别对ICIs的肝脏、胃肠道不良反应的发生率、临床表现、诊断和处理分别进行阐述。

## Abstract (en)

Immunotherapy for malignant tumors is a hot spot in current research and treatment of cancer. The activation of programmed cell death receptor-1 (PD-1) and cytotoxic T lymphocyte-associated antigen 4 (CTLA)-4 relevant signaling pathway can inhibit the activation of T lymphocytes. Tumor cells can achieve immune escape by activating this signaling pathway. By inhibiting this signaling pathway, immune-checkpoint inhibitors (ICIs) activate T lymphocytes to clear the tumor cells. Therefore, the adverse effects of ICIs are mainly immune-related adverse events (irAEs). The digestive system, including gastrointestinal tract and liver are vital organs of digestion and absorption, metabolism and detoxification, as well as important immune related organs, which are the commonly affected system of irAEs. This review separately explains the incidence, clinical features, diagnosis and treatment of liver and gastrointestinal adverse events in ICIs.

## 1 免疫检查点抑制剂（immuno-checkpoint inhibitors, ICIs）的肝脏不良反应

### 1.1 发生率

免疫相关肝毒性的发生率单药治疗（ipilimumab, nivolumab, pembrolizumab）约5%-10%，其中3级约1%-2%。联合治疗（如ipilimumab联合nivolumab）的发生率为25%-30%，其中3级约15% ^[1, 3]^。单药治疗（nivolumab）3级-4级肝毒性的发生时间是14.1周（1.9周-25.1周），联合治疗（如ipilimumab联合nivolumab）3级-4级肝毒性的发生时间提前，平均为7.4周（2.1周-48周），并且持续时间延长^[3]^。

### 1.2 诊断

irAEs肝毒性的发生通常隐匿，可不伴随明显的临床表现，用药后定期监测肝功能有助于早期发现。一旦出现肝功能异常，或较用药前水平上升，需尽早完善包括血生化、肝炎病毒检测、肝脏影像检查，必要时肝活检等一系列检查。通常在肿瘤患者药物治疗前，需完善慢性肝病病史的询问，如饮酒史、长期用药史、慢性肝炎病史、自身免疫性肝病病史等。用药前应筛查自身免疫性肝病相关抗体、病毒性肝炎如乙型、丙型肝炎病毒等。当治疗后出现肝功能异常，需重复上述肝炎病毒血清学检查，以及甲肝、戊肝血清学检查，必要时检测病毒载量（如HBV-DNA、HCV-RNA）。还需常规完善机会性感染病原体，包括巨细胞病毒（Cytomegalovirus, CMV）、人类疱疹病毒第四型（Epstein-Barr virus, EBV）的相关检查。除上述病毒性肝炎、感染、酒精、其他合并药物等因素后，还需考虑肿瘤本身肝脏受累，治疗前的肝脏影像学评估对判断病因有一定帮助。以胆汁淤积（即胆红素升高，以直接胆红素为主，伴随GGT、ALP升高）为主要表现的患者，还需要行腹部影像学（如超声、MRCP）等除外胆道结石、肿瘤等梗阻性因素。

上述病因的鉴别应尽可能及早完善，强调用药治疗前的全面评估，以保证治疗过程中以最短的时间明确诊断，以免延误治疗时机。

对于不良反应严重程度分级为4级（表 1）的患者，需考虑行肝活检，以判断预后。肝穿刺活检病理最常见的表现是小叶性肝炎，与自身免疫性肝炎无法鉴别。大多数病例为广泛小叶病变，如有窦组织细胞增生和中央静脉内皮炎的表现有助于ipilimumab相关炎症的诊断。罕见病例表现为门静脉炎症和胆管炎^[4]^。

**1:** irAEs肝毒性的评估和处理 Evaluation and management of liver toxicity of irAEs

Severity	ALT/AST	Management	Evaluation
G1	< 3 × upper limit of normal (ULN)	Continue ICIs	Monitor liver function tests (LFTs) in 1 week
G2	3-5 × ULN	Hold ICIs Consider prednisone 0.5 mg/kg/d-1 mg/kg/d	Monitor LFTs every 3 days Discontinue concurrent medicine Limits/discontinue hepatotoxic medications (eg. antibiotics, statins, alcohol use, etc) Rule out viral etiology, disease-related hepatic dysfunction Consider abdominal ultrasound
G3	5-20 × ULN	Discontinue ICIs If ALT/AST < 400 and normal TBIL/INR/albumin, consider prednisonlone 1 mg/kg/d If ALT/AST > 400 or abnormal TBIL/INR/albumin, initiate Ⅳ methylpredinisolone 2 mg/kg/d	Evaluation as above Monitor LFTs everyday Consider abdominal ultrasound Hospitalization
G4	> 20 × ULN	Permanently discontinue ICIs Initiate Ⅳ methylpredinisolone 2 mg/kg/d	Evaluation as above Consider hepatologist consultation and liver biopsy
ALT: alanine aminotransferase; AST: aspartate aminotransferase; ICIs: immune-checkpoint inhibitors; LFTs: liver function tests; ULN: upper limit of normal; Ⅳ: intravenous; irAEs: immune-related adverse events.			

### 1.3 处理

肝毒性的处理原则见表 1，按照肝功能ALT/AST的升高水平和是否存在胆红素/INR/白蛋白的异常进行分级。通常G1无需停药；G2根据肝功能好转情况，可考虑择期恢复ICIs治疗；G3/G4需考虑永久性停药。激素是治疗肝毒性的主要药物。如分级为G3/G4，激素治疗有效，肝功能降至G2水平，可由静脉甲强龙转换为口服泼尼松龙，之后约4周逐渐减停。如G2，口服泼尼松龙治疗有效，可在2周内减停。在激素治疗的过程中，肝功能仍进行性加重，需考虑升级强化治疗。如：口服泼尼松龙转换为静脉甲强龙，静脉激素治疗2 d-3 d无效，加用口服麦考酚酸脂（mycophenolic acid, MMF）500 mg-1, 000 mg *bid*；如MMF无效，可考虑加用他克莫司。也有采用抗胸腺细胞球蛋白（anti-thymocyte globulin, ATG）治疗MMF无效的爆发性肝炎的病例报道。ICIs相关肝毒性通常在4-6周恢复，对于持续不缓解的患者，需警惕其他因素导致的肝功能异常，再次排查其他病因，特别需要警惕其他药物、机会性感染（如CMV）等病因。

## 2 ICIs的消化道不良反应

### 2.1 发生率

消化道是irAEs最常见的受累部位。不同作用靶点的消化道irAEs发生率不同，CTLA-4单克隆抗体消化道不良反应发生率高于PD-1单克隆抗体^[6]^。比较PD-1抗体治疗黑色素瘤、非小细胞肺癌、肾细胞癌的irAEs发生率，显示黑色素瘤患者的消化道不良反应发生率更高，肺炎的发生率较低。提示同一药物在不同的免疫微环境中可能驱动组织学特异性的irAEs模式^[6]^。

CTLA-4单克隆抗体治疗肿瘤患者腹泻的发生率为27%-54%。腹泻的发生率约占1/3，结肠炎的发生率为8%-22% ^[7]^。消化道不良反应是CTLA-4单克隆抗体治疗最常见的irAEs，也是最重且最早出现的，常导致药物停用。有研究报道，1%-1.5%黑色素瘤接受ipilimumab治疗的患者发生结肠穿孔，肾细胞癌患者发生穿孔的比例高达6.6% ^[8]^。PD-1单克隆抗体发生3级/4级消化道irAEs的比例为1%-2% ^[1]^。

CTLA-4抗体消化道irAEs可出现在第1-10次用药的任何时间，甚至可在末次用药后数月出现^[8]^。消化道irAEs发生中位时间是7.4周（1.0周-48.9周）^[3]^。CTLA-4抗体与PD-1抗体联合应用消化道irAEs的发生率更高，程度更重，且发生时间更早^[1]^。

消化道irAEs的高危因素包括服用NSAIDs药物，有炎症性肠病病史等^[9, 10]^。

### 2.2 诊断

消化道irAEs最常见的表现是腹泻，其他包括腹痛、便血、恶心、呕吐、体重下降、发热等。可同时伴随多种肠道外受累表现，如关节痛、内分泌异常、皮肤损害、肝炎、肾炎、心包炎、胰腺炎等irAEs。实验室检查可出现C反应蛋白升高、贫血、低白蛋白血症，少部分患者可出现自身免疫性抗体如抗中性粒细胞胞浆抗体（antineutrophil cytoplasmic antibody, ANCA）等阳性^[9]^。内镜下表现多为左半结肠受累，黏膜充血、血管纹理消失、糜烂和溃疡，病变可弥漫分布，也可呈不连续性分布。组织学特点常表现为急性损伤（如中性粒细胞、嗜酸性粒细胞浸润），局灶或弥漫，可有隐窝脓肿。一部分患者可呈现组织学上慢性炎症如隐窝结构紊乱（分支、萎缩、出芽、扭曲等）、基底部浆细胞增多甚至肉芽肿表现^[9]^。消化道irAEs和消化道一类慢性非特异性疾病即炎症性肠病（inflammatory bowel disease, IBD）的临床表现、内镜表现、甚至病理特点均有相似、重叠之处，表 2总结了消化道irAEs的临床、内镜、病理和发病机制等特点，以及与IBD的可鉴别之处^[11]^。

**2:** 消化道irAEs的临床、内镜、病理特点总结 Summary of clinical, endoscopic and pathological features of gastrointestinal irAEs

	Gastrointestinal irAEs
Clinical features	Acute onset Mild to life-threatening diarrhea, bowel perforation in severe patients Severity of gastrointestinal differs in different ICIs Higher risk in patients with a medical history of inflammatory bowel disease
Endoscopic features	Diverse endoscopic manifestations; Rectum often spared; left colon often involved; diffuse lesion or segmental lesions
Pathological features	IBD-like (increased basal plasma cells, crypts and apoptotic bodies are more common) Lymphocytic colitis-like Celiac disease-like (mostly seen in upper gastrointestinal tract) GVHD like
Immune changes	Clear immune pathogenesis CD4^+^ based T lymphocyte proliferation Th1/Th17 up-regulation interaction with intestinal microbiota
IBD: inflammatory bowel disease; GVHD: graft versus host disease.	

消化道irAEs的诊断有赖于出现上述临床症状与ICIs用药的时间关系，具有上述实验室检查、内镜、组织病理学特点。同时，需除外其他病因，包括：感染性肠炎，如艰难梭菌感染、CMV结肠炎等机会性感染；缺血性结肠炎；其他药物导致的结肠炎，如非甾体抗炎药（nonsteroidal anti-inflammatory drugs, NSAIDs）等；放射性肠炎等。因此，建议完善粪便病原学检查（便常规、便培养、便艰难梭菌毒素检测、粪便寄生虫检测等）、血CMV-DNA等病毒相关病原学检测；同时建议完善腹盆部增强CT；经消化科医生会诊后完善消化内镜并内镜下组织活检。

### 2.3 处理

消化道irAEs的处理原则是尽早识别、及时足量治疗、快速升级、改善预后。根据腹泻的次数进行严重程度分级，给予分层治疗。表 3详细列举不同严重程度消化道irAEs的处理方案。糖皮质激素是中重度消化道irAEs的主要治疗，如中度患者治疗有效，激素可在2周-4周减停；重度患者可在4周-8周减停。如激素治疗效果不佳，需及时调整激素剂量/剂型，必要时快速升级至英夫利昔单抗（infliximab, IFX）或维多株单抗。研究显示，与长期激素治疗比较，短期激素联合IFX治疗消化道irAEs合并各种感染的风险降低^[12]^。对于激素、IFX、维多株单抗均无效的难治性消化道irAEs，有病例报道显示肠道菌群移植治疗有效^[13]^。

**3:** 消化道irAEs的评估和处理 Evaluation and management of gastrointestinal irAEs

Severity	Management	Evaluation
Mild（G1）: fewer than 4 bowel movements per day above baseline	Continue ICIs Symptom control: hydration, loperamide Avoid high fiber/lactose diet	Stool evaluation to rule out infectious etiology: Clostridium difficile, CMV, etc
Moderate (G2): 4-6 bowel movements above baseline per day, colitis symptom (bloody diarrhea, abdominal pain)	Hold ICIs Prednisone 0.5 mg/kg/d-1 mg/kg/d No response in 48-72 hours, increase dose to 2 mg/kg/d	Evaluation as above GI consultation Schedule colonoscopy/sigmoidoscopy Recheck above tests every 3 days
Severe (G3/4): more than 6 bowel movements above baseline per day, other serious complications (eg. Ischemic bowel, perforation, toxic mega-colon)	Discontinue ICIs Hospitalization Consider NPO, supportive care Ⅳ methylprednisolone 1 mg/kg/d-2 mg/kg/d No response in 48 hours, continue steroids, consider adding IFX If IFX refractory, consider vedolizumab	Evaluation as above Consider abdominal/pelvic CT with contrast Monitor complete blood count, liver and kidney function tests, electrolytes, and C-reactive protein every day
CMV: cytomegalovirus; Ⅳ: intravenous; IFX: infliximab; NPO: nothing by mouth.		

## 3 肠道菌群与消化道irAEs及肿瘤预后

目前尚未发现可预测消化道irAEs的生物标志物。最新的研究探索，ICIs治疗前基线的粪便微生物群组成可预测ipilimumab诱导的结肠炎。研究显示，基线时富含梭菌属和其他厚壁菌门的肠道微生物群与ipilimumab的治疗效果好相关，同时免疫相关结肠炎发生率高^[14]^。2018年初发表于*Science*上的多项研究提示治疗前特定的粪便微生物群组成与肿瘤对于ICIs的治疗反应相关^[15, 16]^。这将为进一步提高ICIs治疗肿瘤的疗效、改善预后带来前景。

## 4 总结

伴随ICIs在肿瘤治疗中的广泛应用，irAEs也越来越被肿瘤科医生以及所涉及的各个专科医生所重视。消化系统以消化道受累（腹泻/结肠炎）最为突出，其次是肝脏受累。irAEs的基线筛查、早期识别、及时诊断和快速足量的治疗是解决该类临床问题的关键。肠道微生物群组成是否能预测消化道irAEs以及预测ICIs治疗肿瘤的预后值得进一步的研究探索。
